# Differentially expressed transcript isoforms associate with resistance to tuberculin skin test and interferon gamma release assay conversion

**DOI:** 10.1371/journal.pone.0284498

**Published:** 2023-04-14

**Authors:** Jason D. Simmons, R. Max Segnitz, Kimberly A. Dill-McFarland, Catherine M. Stein, Glenna J. Peterson, Harriet Mayanja-Kizza, W. Henry Boom, Thomas R. Hawn

**Affiliations:** 1 TB Research & Training Center, Department of Medicine, University of Washington, Seattle, Washington, United States of America; 2 Department of Population & Quantitative Health Sciences, Case Western Reserve University, Cleveland, Ohio, United States of America; 3 Department of Medicine, School of Medicine, Makerere University, Kampala, Uganda; 4 Department of Medicine, Case Western Reserve University, Cleveland, Ohio, United States of America; Shandong Public Health Clinical Center: Shandong Provincial Chest Hospital, CHINA

## Abstract

**Background:**

A mechanistic understanding of uncommon immune outcomes such as resistance to infection has led to the development of novel therapies. Using gene level analytic methods, we previously found distinct monocyte transcriptional responses associated with resistance to *Mycobacterium tuberculosis* (Mtb) infection defined as persistently negative tuberculin skin test (TST) and interferon gamma release assay (IGRA) reactivity among highly exposed contacts (RSTR phenotype).

**Objective:**

Using transcript isoform analyses, we aimed to identify novel RSTR-associated genes hypothesizing that previous gene-level differential expression analysis obscures isoform-specific differences that contribute to phenotype.

**Materials and methods:**

Monocytes from 49 RSTR versus 52 subjects with latent Mtb infection (LTBI) were infected with *M*. *tuberculosis* (H37Rv) or left unstimulated (media) prior to RNA isolation and sequencing. RSTR-associated gene expression was then identified using differential transcript isoform analysis.

**Results:**

We identified 81 differentially expressed transcripts (DETs) in 70 genes (FDR <0.05) comparing RSTR and LTBI phenotypes with the majority (n = 79 DETs) identified under Mtb-stimulated conditions. Seventeen of these genes were previously identified with gene-level bulk RNAseq analyses including genes in the IFNγ response that had increased expression among LTBI subjects, findings consistent with a clinical phenotype based on IGRA reactivity. Among the subset of 23 genes with positive differential expression among Mtb-infected RSTR monocytes, 13 were not previously identified. These novel DET genes included *PDE4A* and *ZEB2*, which each had multiple DETs with higher expression among RSTR subjects, and *ACSL4* and *GAPDH* that each had a single transcript isoform associated with RSTR.

**Conclusion and limitations:**

Transcript isoform-specific analyses identify transcriptional associations, such as those associated with resistance to TST/IGRA conversion, that are obscured when using gene-level approaches. These findings should be validated with additional RSTR cohorts and whether the newly identified candidate resistance genes directly influence the monocyte Mtb response requires functional study.

## Introduction

Exposure to *Mycobacterium tuberculosis* (Mtb) results in diverse outcomes including tuberculosis disease (TB), asymptomatic infection with risk of progression to TB, and clearance of the pathogen through innate and/or cellular immune responses. The host-pathogen determinants that result in clearance of the bacillus are poorly understood, in part due to the limitations in microbiologically defining these phenotypic outcomes. Clinically, the term latent Mtb infection (LTBI) currently describes asymptomatic and TB immunoreactive subjects as defined by the interferon gamma (IFNγ) release assay (IGRA) or the tuberculin skin test (TST). While TB immunoreactivity is an imperfect surrogate for Mtb infection [1], subjects with heavy exposure who resist TST/IGRA conversion (RSTR phenotype) offer an opportunity to characterize Mtb immune responses that correlate with this alternative immune outcome. We reported that longitudinal TST/IGRA non-reactivity among highly-exposed Mtb contacts in Uganda is not explained by a lack of Mtb exposure, which has been confirmed using both rigorous epidemiologic characterization [2, 3] and Mtb antigen-specific T cell responses that are independent of IFNγ [4]. While the negative predictive value of TST and IGRA for the risk of TB progression is well-established and exceeds 98–99% [5–7], it is currently unknown whether resistance to TST/IGRA conversion after heavy exposure *per se* translates to protection from TB progression considering the practical challenges of recruitment and longitudinal follow-up of these cohorts. However, a better understanding of the unique immune responses of stringently defined RSTR cohorts may uncover novel immune pathways that are critical for control of Mtb.

We previously correlated monocyte transcriptional responses to ex vivo Mtb infection among heavily exposed household contacts in Uganda and identified distinct inflammatory signatures that correlate with clinical resistance to TST/IGRA conversion [8]. For that analysis, transcript reads were aligned to exons and summed to a gene-level count table before differential gene expression analysis. Although this is a standard pipeline for microarray and bulk RNAseq differential expression analysis [9], condensing diverse transcript isoforms to the gene level ignores the dynamics of alternative splicing and may obscure transcript isoform-specific associations with phenotypes. Various open-access tools exist to assign transcript abundance estimates (e.g. Kallisto [10]) and to perform differential transcript isoform expression analysis, (e.g. Sleuth [11]).

In the current study, we revisit the monocyte transcriptional response to Mtb with a focus on transcript isoform expression using Sleuth. We describe previously unrecognized RSTR-associated responses including differentially expressed transcripts (DETs) with plausible biologic contributions toward resistance to TST/IGRA conversion. These findings contribute to the growing literature that aims to define the underpinnings of the uncommon RSTR immune outcome and provides targets for future mechanistic experiments.

## Materials and methods

### Study aims, design and participant recruitment

We aimed to identify transcriptional responses that distinguish monocytes isolated from participants with LTBI (TST+ and IGRA+) from those who resist TST/IGRA conversion after heavy exposure and determine whether differential transcript isoform analysis would yield novel findings as compared to previous gene-level differential expression analysis [8]. The recruitment of participants was part of a parent study that has been described in detail [2, 3] including the study protocol that was approved by the institutional review board at University Hospitals Cleveland Medical Center, the National HIV/AIDS Research Committee of Makerere University and the Uganda National Council for Science and Technology. In brief, HIV-negative subjects who provided written consent were initially recruited as part of the Kawempe Community Health Study [3] and assigned a clinical phenotype based on longitudinal TST/IGRA testing. This included serial TST testing during the two years immediately after household exposure (phase 1; 2002–2012) followed by a retracing study (phase 2; 2014–2017) when three IGRA tests and a single TST were performed [2]. For the current study, RSTR subjects were defined as those with negative TST and IGRA test results from all sampling times whereas LTBI subjects were those with positive TST and IGRA test results at all available times (e.g. concordant ‘definite’ criteria [2]). Importantly, there were no differences in age, sex, BMI, BCG scar prevalence, exposure score or relatedness by kinship according to RSTR versus LTBI phenotype among the subjects included in this monocyte transcriptional profiling substudy (n = 49 RSTR and 52 LTBI) [8]. Cryopreserved peripheral blood mononuclear cells (PBMCs) were isolated from these subjects, shipped and the following *ex vivo* experiments were performed with approval from the University of Washington Institutional Review Board (STUDY00001537).

### CD14+ monocyte isolation, Mtb culture and stimulation

CD14+ monocytes were isolated from PBMCs by magnetic bead column isolation (Miltenyi) and cultured in RPMI-1640 (Gibco) supplemented with 10% fetal bovine serum (Atlas Biologicals) and monocyte colony stimulating factor (M-CSF, Peprotech) for a total of 48 hours before Mtb stimulation as previously described [8, 12]. In a BSL3 laboratory, Mtb H37Rv (gift of David Sherman) was cultured to log-phase in 7H9 media supplemented with glycerol and albumin-dextrose-catalase (Gibco), washed and reconstituted in Sauton’s media to prepare frozen stocks. Freshly thawed H37Rv stocks were diluted in complete RPMI media to the desired optical density, applied to monocyte cultures (multiplicity estimated at 1 CFU per cell) in singlet along with media-only controls, centrifuged (~300 x g x 5 minutes) to maximize phagocytosis, and incubated at 37 °C/5% CO2 for 6 hours. Stimulations were done in singlet (media and Mtb condition) for each subject, but the experiment was performed twice with partially overlapping subjects as described [8] totaling 117 (58 RSTR and 59 LTBI) paired transcriptomes (media and Mtb-stimulated) from 49 RSTR and 52 LTBI unique subjects.

### RNA isolation, sequencing and transcript isoform differential expression analysis

As previously described in detail [8, 12], RNA was isolated from monocytes using miRNeasy micro columns (Qiagen), quality was ensured (RIN ≥ 8.0) by Agilent TapeStation, and cDNA libraries were prepared after rRNA depletion using random hexamer primers (Takara). Sequences from the Illumina Hiseq 2500 (Experiment A) or Novaseq 6000 (Experiment B) were aligned to GRCH38 reference genome (STAR2.6.0a) with counts assigned using RSEM 1.3.0. After filtering and quality control, gene-level differential expression analysis was performed with lmekin in the R package coxme [13, 14] as described in detail [8]. For isoform level analysis, RNAseq reads were instead mapped by pseudoalignment to the GENCODE v34 transcriptome [15] using Kallisto v0.46.1 [10] including sequence based bias correction, a sample bootstrapping specification of ‘50’, and all other default settings. Rare transcripts were removed from transcript abundance tables retaining those with at least 5 reads in at least 60% of samples; this removed 174,399 transcripts yielding 53,649 transcripts for statistical analysis. We tested for differential expression of transcripts using Sleuth v0.30.1 [11], which incorporates bootstrapped estimates of technical variance into linear modeling of transcript expression. Sigma plots indicated that correction for sequencing batch improved the model fit for ~80% of transcripts, and thus we included batch as a covariate in our models along with correction for age and sex (S1 Fig). We assessed differential transcript expression across phenotypes (RSTR—LTBI) in the media condition and separately in the Mtb-stimulated condition, adjusted for age, sex and sequencing batch. A secondary, gene-level analysis limited to genes with at least one DET (FDR <0.05) was performed in lmekin using identical formulation as the Sleuth transcript models, with an additional adjustment for kinship as a random effect. We compared results from the Sleuth isoform-specific analysis and the gene-level lmekin secondary analysis with the differentially expressed genes identified previously that used an expression model (Mtb:RSTR) that incorporates an interaction term with the main effects: Expression ~ phenotype + stimulation + phenotype:stimulation + covariates (age, sex, sequencing batch) with patient and genetic kinship included as random effects.

### STRING network analysis

Differentially expressed transcripts (DETs) from the media (n = 2) and Mtb-stimulated (n = 79) conditions yielded 67 genes that were analyzed by STRING [16]. Edges indicate both functional and physical protein associations using a minimum combined score of 0.4 from all available sources (protein-protein interactions from experimental and curated databases, gene neighborhood and gene fusion, co-occurrence, co-expression, and text mining).

### Inclusivity in global research

Additional information regarding the ethical, cultural, and scientific considerations specific to inclusivity in global research is included in the S1 Checklist.

## Results

**Fig 1:**
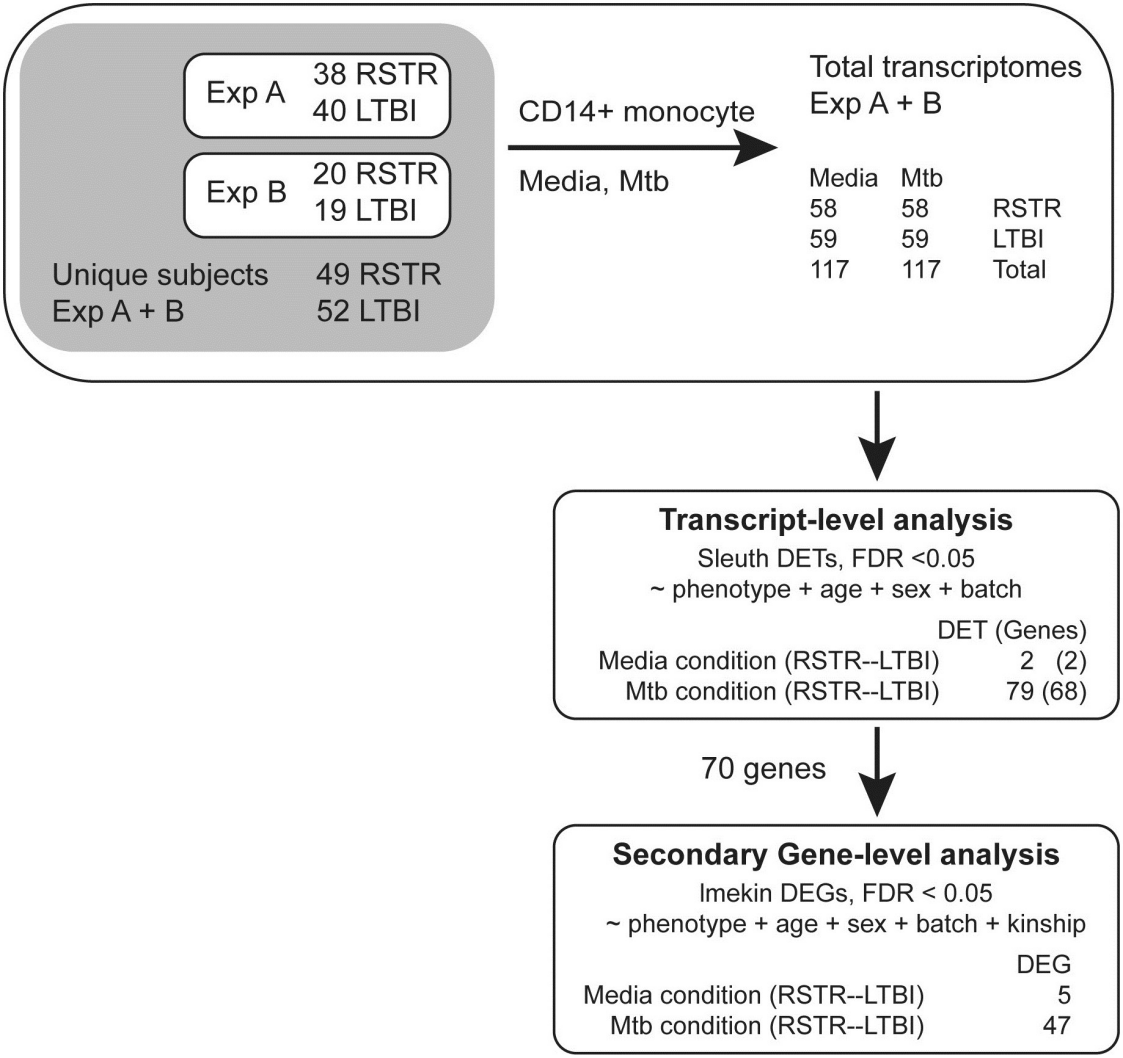
Experimental outline of donor monocyte infections, isoform-specific and gene-level differential expression analysis for the RSTR—LTBI contrast. Two independent experiments (Experiment A and B) were performed with CD14+ monocytes from the indicated subjects with partial overlap totaling 58 and 59 transcriptomes from 49 and 52 unique RSTR and LTBI subjects, respectively, in each condition (media or Mtb-stimulated). Primary transcript isoform differential expression analysis was performed using Sleuth, and among 68 genes with at least one differentially expressed transcript (DET, false discovery rate [FDR] <0.05), a secondary gene-level analysis using lmekin that adjusts for genetic kinship was performed.

To identify genes and pathways that may distinguish RSTR and LTBI clinical phenotypes, we previously used a traditional bulk RNAseq analytic approach of Mtb infected monocytes in which all reads were aligned to a reference genome to generate gene-level counts [8]. In that study, monocytes were infected with Mtb (1 CFU per cell) or left uninfected (media) for 6 hours after which RNA was isolated for sequencing. In total, 117 transcriptomes for each stimulation condition were analyzed from 101 unique subjects (49 RSTR and 52 LTBI) among whom we previously found no epidemiologic or demographic differences according to clinical phenotype (Fig 1).

**Fig 2:**
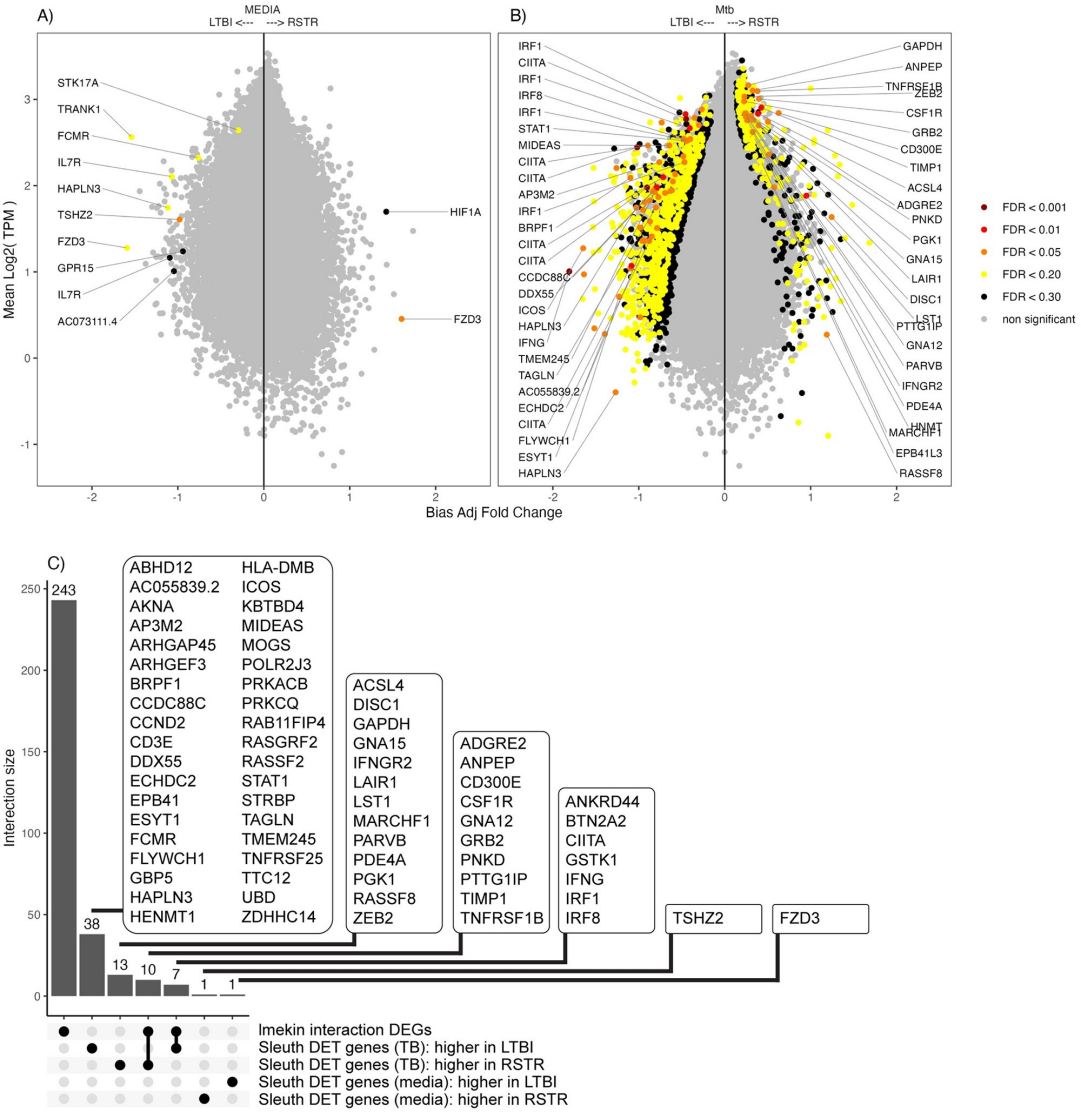
Differential (RSTR—LTBI) transcript isoform expression in media and Mtb-stimulated conditions. For the media (A) and Mtb-stimulated (B) conditions, overall expression (y-axis; log2 transcript count per million [TPM]) is plotted against transcript fold change expression (RSTR—LTBI) where isoforms with positive fold change indicate higher average expression among RSTR donors (x-axis, bias adjusted log2 fold change). False discovery rate [FDR] for the RSTR—LTBI contrast for each condition are indicated by color shading. (C) UpSet plot compares overlap of differentially expressed genes (DEGs) identified previously with a gene-level interaction model (Mtb:RSTR lmekin) and genes with ≥ 1 DET organized according to the indicated subsets.

While gene-level bulk RNAseq analysis is robust and captures meaningful differences in the major mRNA isoforms, we hypothesized that additional biologic insight into these clinical phenotypes could be identified by comparing each transcript isoform individually using Sleuth [11]. After correction for multiple comparisons, only two differentially expressed transcripts (DETs) were identified in unstimulated cells (false discovery rate [FDR] < 0.05; Fig 2A, S1A Table), which is similar to our previously reported findings that identified zero gene-level differences between these clinical groups in the unstimulated condition [12]. However, after Mtb-stimulation, 79 DETs in 68 genes distinguished RSTR and LTBI phenotypes (FDR <0.05; Fig 2B, Table 1 and S1B Table).

**Table 1:** RSTR versus LTBI differentially expressed transcripts (DETs), Mtb condition.

	No. DETs by false discovery rate (FDR) cutoff			
	<0.05	<0.1	<0.2	<0.3
Media	2	5	8	12
Mtb	79	168	867	1723

Among DETs with higher expression following Mtb infection in LTBI monocytes were transcripts for *CIITA*, *STAT1*, *IFNG*, *IRF1* and *IRF8* (S1B Table), each of which relate to the cellular IFNγ response and is an expected finding based on our previous analysis [8] and the IGRA-defined LTBI phenotype definition. Importantly, we also identified DETs in 51 genes that had not been previously identified using gene-level differential gene expression (Fig 2C) [8]. Among these newly identified genes, the subset with higher DET expression in RSTR monocytes included *ACSL4*, *LST1*, *GAPDH*, *ZEB2* and *PDE4A*. Furthermore, the top RSTR DET *TIMP1* (log2FC 0.425, FDR 0.005) had previously been identified among RSTR DEGs but had a much lower gene rank (145 of 260 DEGs, FDR 0.143) [8]. We also noted that STRING network analysis [16] of the 70 DET genes connected ZEB2 and PDE4A to a larger network via interactions with DISC1 and IRF8, respectively (S2 Fig).

**Fig 3:**
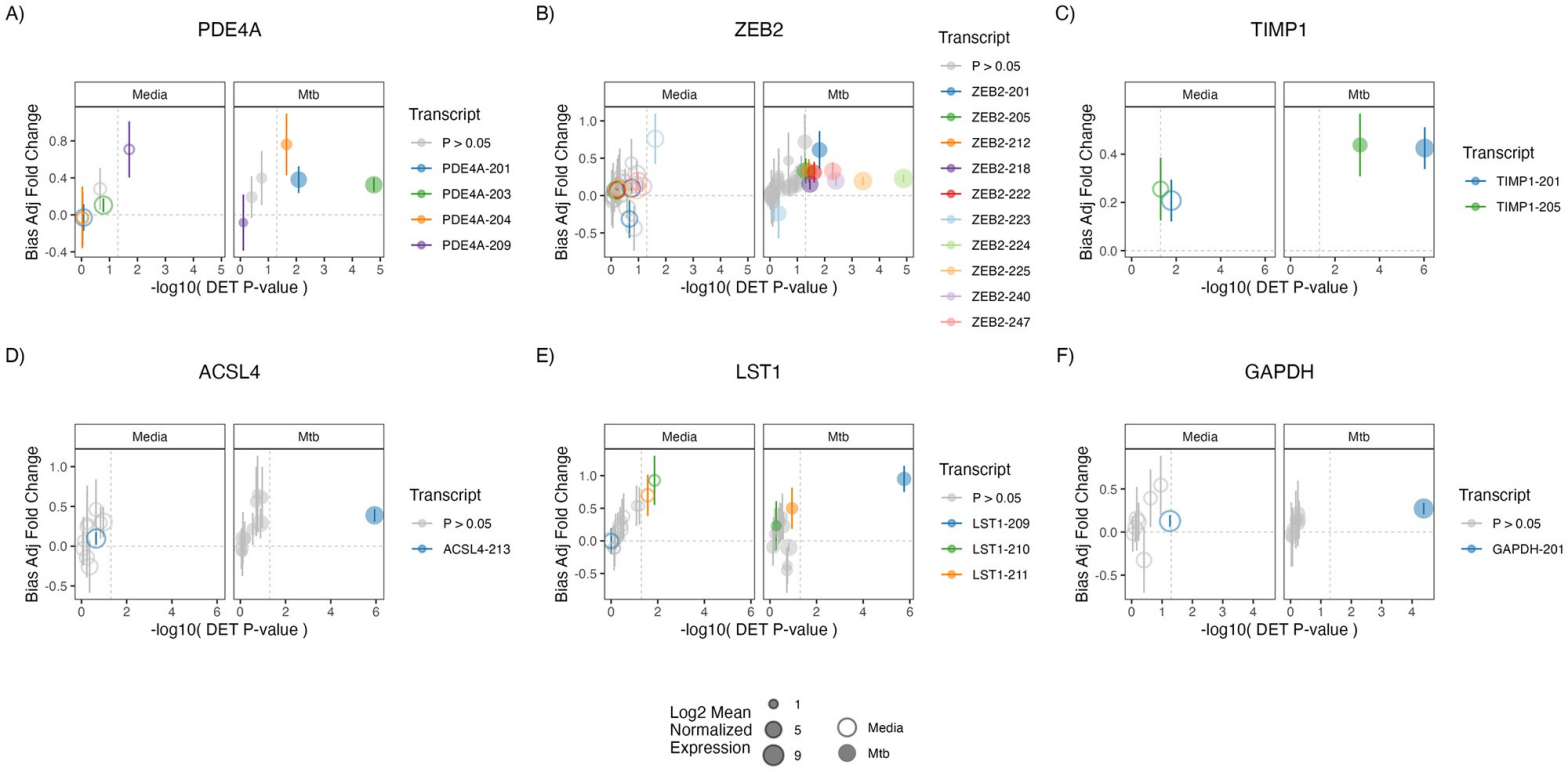
Transcript isoform specific relative expression reveals unique and conserved expression patterns across newly identified genes with RSTR-associated DETs. For the indicated genes with at least one DET of interest, each transcript isoform is plotted as a node according to RSTR—LTBI fold-change with standard error and P value. Significant DETs (P <0.05, vertical dashed line) are indicated by paired colors to compare media (unfilled) and Mtb-stimulated (filled) conditions. Log2 mean normalized expression is indicated by size of transcript isoform node.

To further explore transcript expression patterns of these DETs of interest, we contrasted fold-change (RSTR versus LTBI) expression for each DET and other transcripts within that gene and noted two patterns (Fig 3). First, among DET genes in the Mtb condition, transcript expression was consistently increased among RSTR donors for genes like *PDE4A*, *ZEB2* and *TIMP1* (Fig 3A–3C). Second, some genes like *ACSL4*, *GAPDH* and *LST1* (Fig 3D–3F) had a single DET with highly significant RSTR fold-change while other transcripts in that gene had inconsistent or weak associations with phenotype. The DETs ACSL4-213 (log2FC 0.39, FDR 0.006) and GAPDH-201 (log2FC 0.27, FDR 0.042) are the major protein-coding Ensembl canonical transcripts for their respective genes, whereas the LST1-209 variant (log2FC 0.95, FDR 0.008) encodes a severely truncated 13 amino acid isoform raising questions of its biologic importance. In summary, although *ACSL4* and *GAPDH* each encode only a single transcript variant that is significantly increased among RSTR subjects, the differential expression of these isoforms is expected to translate to differences in protein levels. These differences detected by differential transcript analysis may contribute to the divergent phenotypic outcome (e.g. TST/IGRA conversion) and are obscured using gene-level analysis.

**Fig 4:**
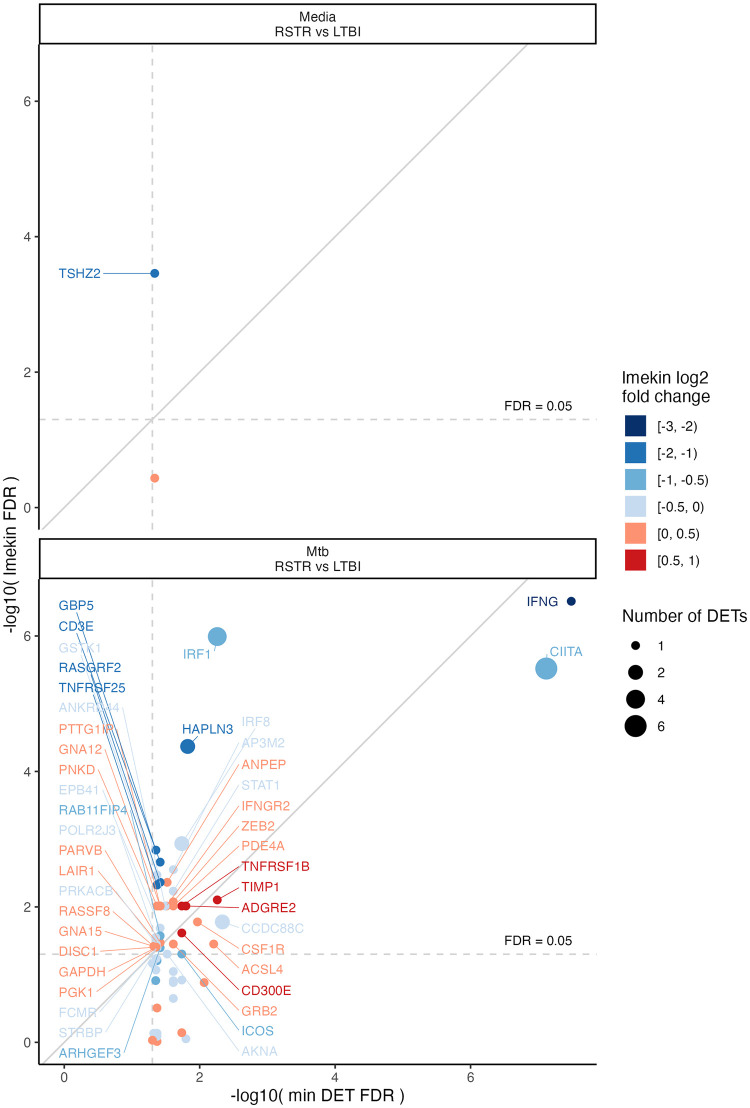
Secondary analysis of genes with ≥1 DET indicates the majority remain kinship-adjusted DEGs. Genes with ≥1 DET (FDR<0.05; 65 from Mtb and 2 from media condition) were analyzed by lmekin in the media (top) and Mtb-stimulated (bottom) conditions to confirm which RSTR—LTBI differentially expressed genes (DEGs) remained significant after adjustment for genetic kinship. Correlation plots compare the FDR for RSTR—LTBI expression at the gene-level (lmekin model, y-axis) and transcript level (DET with lowest Sleuth model FDR, x-axis). Node size indicates the number of DETs (FDR <0.05), and color indicates gene-level lmekin log2 fold change in expression.

To address possible confounding effects of relatedness within the participant cohort, we performed a secondary gene-level analysis to adjust for kinship. Because Sleuth is unable to account for a covariance matrix random effect such as kinship, we analyzed genes with at least one RSTR versus LTBI DET (media or Mtb condition; FDR <0.05) and performed kinship-adjusted differential gene expression analysis (RSTR versus LTBI comparison) using lmekin for the media or Mtb condition. The majority (47 of 67) of these genes remained DEGs (Table 2), and among genes with DETs having higher expression among RSTR subjects, 20 of 22 remained kinship-adjusted DEGs including *PDE4A*, *ZEB2*, *TIMP1* and *ACSL4* (FDR <0.05). A correlation between the FDR for each DET and its corresponding RSTR DEG incorporating these adjustments identifies IFNG, CIITA, IRF1, HAPLN3, IRF8, TIMP1, ZEB2 and PDE4A among the most significant hits that are robust to both analyses and suggests they have the strongest association with biologic differences in the monocyte Mtb response between LTBI and RSTR donors (Fig 4). Overall, our secondary analysis suggests that the majority of DETs, even those that are unique among other transcript isoforms for a given gene, also represent gene-level RSTR—LTBI associations even after kinship adjustment and supports the inclusion of these genes in further analyses.

**Table 2:** Among genes with ≥1 DET (FDR <0.05), number of RSTR versus LTBI differentially expressed genes (DEGs) by condition and false discovery rate using lmekin.

	False discovery rate (FDR)			
	<0.05	<0.1	<0.2	<0.3
Media	5	5	11	22
Mtb	47	53	59	60
Total non-redundant	47	53	59	61

## Discussion

We explored whether differential transcript isoform expression analysis provides additional biologic insight into mechanisms of TST/IGRA resistance. Compared to a previous gene-level analysis of monocyte bulk RNAseq data, our transcript level analysis yielded highly consistent results including higher transcript-specific expression of genes involved with IFNγ and inflammatory signaling (e.g. *IFNG*, *CIITA*, *IRF1*, *STAT1*) among subjects with LTBI. Yet 51 of the 68 genes with at least one DET were not previously identified using gene-level analysis. Several of these newly identified genes with significantly higher transcript expression among RSTR donors (Sleuth FDR <0.05) included *PDE4A*, *ZEB2*, *ACSL4*, *LST1* and *GAPDH*. We conclude that isoform-level analysis identifies genes previously ignored by gene-level analysis but the role that these genes play, if any, during resistance to TST/IGRA conversion must be evaluated experimentally.

Our results suggest several mechanisms that may contribute to the monocyte Mtb response and influence TST/IGRA conversion. By regulating intracellular cyclic nucleotide concentrations, phosphodiesterase 4A (PDE4A) expression in immune cells results in increased inflammatory mediator secretion and is the target of various FDA approved immune modulators [17]. In a pre-clinical animal model [18, 19] and a recent human randomized clinical trial [20, 21], PDE4 inhibition limited immunopathology and improved pulmonary function among subjects with TB. In contrast to a potentially detrimental effect at late timepoints, the expression of multiple *PDE4A* isoforms was higher among RSTR subjects immediately following ex vivo Mtb infection suggesting an early hyperinflammatory response may be protective for TST/IGRA conversion. STRING analysis also linked PDE4A to other DETs including PRKACB and DISC1 as a regulator of intracellular cAMP levels and by physical interaction, respectively [22]. DISC1 is also a scaffolding protein responsible for recruiting the DET GRB2 to neuronal axon tips [23]. Zinc finger E-box-binding homeobox 2, encoded by *ZEB2*, is a transcriptional repressor recently linked to hypoxia-inducible factor-1α (HIF1α) signaling [24]. ZEB2 gene targets include *HDAC2* and expression has been linked to tumorigenesis [25] and regulation of differentiation of specific monocyte, dendritic cell and T cell populations [26–28]. Network analysis of DETs using STRING linked ZEB2 and IRF8, which in mice drive differentiation of distinct tissue macrophage and DC populations [29]. Interestingly, genome-wide linkage analysis associated a locus on chromosome 2 that contains ZEB2 with persistently negative TST responses among exposed household contacts [30] providing orthogonal genetic support for a role ZEB2 may play in the Mtb immune response, but a specific mechanism is not currently known.

Interestingly, among our top DETs with increased RSTR expression were *ACSL4* (RSTR-LTBI contrast in Mtb condition log2FC 0.39, FDR 0.006) and *GAPDH* (log2FC 0.27, FDR 0.04) each of which are regulators of carbon metabolism. Mtb-induced metabolic perturbations within the host macrophage are well described [31–33], but whether these shifts represent host-protective responses or are features of microbial virulence remains uncertain. We previously identified transcriptional programs among unstimulated RSTR monocytes that relate to metabolic programs such as the response to free fatty acids [12]. Acyl-CoA Synthetase Long Chain Family Member 4, encoded by *ACSL4*, catalyzes the formation of long-chain acyl-CoA from free fatty acids (FFAs) targeting them for subsequent synthetic or degradative pathways whereas glyceraldehyde-3-phosphate dehydrogenase, encoded by *GAPDH*, plays a central role in glycolysis and gluconeogenesis while coupling its metabolic activity to NADH synthesis that supports mitochondrial oxidative phosphorylation. Taken together, the DETs identified here with increased expression among RSTR monocytes offer plausible targets for inflammatory and immunometabolic studies to delineate the roles these genes and pathways play in mediating resistance to TST/IGRA conversion.

The identification of new transcripts, or transcripts with stronger RSTR associations compared to our previous gene-level analysis, could reflect biological or methodological differences. The previous gene-level expression model incorporated the interaction between stimulation (Media–Mtb) and phenotype (RSTR–LTBI) to identify DEGs and adjusted for kinship using lmekin. We identified no DETs (FDR <0.05) when incorporating a similar interaction model without kinship since Sleuth does not support matrix random effects (S1C Table). However, by separately assessing each RSTR versus LTBI contrast within each condition (media or Mtb), we uncovered novel RSTR-specific transcripts. Specifically, *PDE4A* and *ZEB2* each had multiple DETs with increased expression among RSTR donors in the Mtb condition whereas these genes were not identified in the lmekin interaction model. The discordant findings may reflect the effects of other transcripts within each gene that minimize the gene-level fold-change or differences in degrees of freedom and power between the two models. Importantly, our kinship-adjusted secondary gene-level analysis (lmekin) found that *ZEB2* and *PDE4A* were among DEGs with the strongest RSTR associations supporting the utility of the contrast model and filtering gene lists using isoform differential expression. In contrast, we also found transcripts such as ACSL4-213 and GAPDH-201 with expression that strongly associated with the RSTR phenotype while all other transcript isoforms within the same genes had no association. Since these transcripts are among the most expressed isoforms in our analysis and are the reference protein-coding transcripts for their respective genes, these expression differences are likely to translate to biological effects and supports the robustness of DET level findings. These genes were also confirmed in our secondary analysis but missed the original gene-level analysis, potentially due to contributions from the non-protein coding transcripts and genome-wide false discovery correction.

Our study has several limitations including its exploratory nature requiring validation either through transcriptional or proteomic profiling of separate RSTR cohorts or through mechanistic studies that confirm these DETs modulate monocyte Mtb responses. We hypothesized that mRNA splice variant analysis would yield novel insight as compared to traditional gene-level differential expression, and while additional DET genes were identified, our overall findings are highly consistent between transcript isoform and gene-level approaches. Whether similar host gene pathways would be identified among RSTR subjects following other Mtb lineages or clinical strains should be considered since our study used only the H37Rv strain (lineage 4) as cell availability from participants was limited. Finally, TB immunoreactivity as defined by TST/IGRA is only a surrogate for Mtb exposure and not Mtb infection, which would require a direct microbiologic diagnostic test that is not currently available. The RSTR and LTBI subjects included in this study show immunologic evidence of Mtb exposure [4] and no demographic or epidemiologic factors distinguished these phenotypes including an exposure risk score [2, 8]. Our findings support growing evidence that RSTR and LTBI subjects have distinct immunologic responses [4, 34–36]. However, larger cohorts of highly exposed Mtb contacts with longitudinal follow-up are required to establish whether these RSTR responses correlate with reduced TB risk, and by extension a higher likelihood of Mtb clearance, or simply represents an alternative immune outcome with similar Mtb infection rates as compared to LTBI.

## Supporting information

S1 Fig Model fit after adjustment for sequencing batch, age and sex. Sigma plots demonstrate improved fit for ~80% of transcripts when the model is adjusted for sequencing batch, whereas only 28% and 37% of transcripts have improved fit when the model is adjusted for age and sex, respectively. All three adjustments were applied to the final model.
(TIF)

S2 Fig STRING network of genes with ≥1 differentially expressed transcript. Genes (n = 67) with ≥1 differentially expressed transcript (DET, FDR <0.05) either from the media (n = 2 DETs) or Mtb-stimulated condition (n = 77 DETs) were analyzed by STRING (string-db.org) using known interactions (experimentally determined or derived from curated databases), co-expression and text mining. One gene (AC055839.2) did not map to STRING. Nodes represent genes. Edges are combined scores >400 (medium confidence) with thickness indicating score value.
(TIF)

S1 Table RSTR—LTBI Differentially expressed transcripts by condition. Differentially expressed transcripts (DETs) for the RSTR—LTBI comparison are listed for the media (S1A worksheet) and Mtb stimulated conditions (S1B worksheet) and ranked by false discovery rate (FDR). Direction indicates whether expression is increased within the RSTR group (up) or LTBI group (down). Genes with ≥1 DET (FDR <0.05) are also noted (Y/N) if they were previously identified [8] as differentially expressed genes (DEGs) by the interaction term Mtb*RSTR using the lmekin interaction model (FDR <0.2). No significant DETs (FDR < 0.05) were identified by incorporating a similar Mtb*RSTR interaction model in Sleuth (S1C) without the kinship term.
(XLSX)

S1 Checklist Inclusivity in global research. PLOS checklist regarding *Ethical considerations*, *permits and authorship* and *Human subjects research* is available.
(DOCX)
