# Supplementary figures and images for: Differentially expressed transcript isoforms associate with resistance to tuberculin skin test and interferon gamma release assay conversion
Source: PLoS One. 2023 Apr 14;18(4):e0284498. doi: 10.1371/journal.pone.0284498 (PMC10104279; doi:10.1371/journal.pone.0284498)

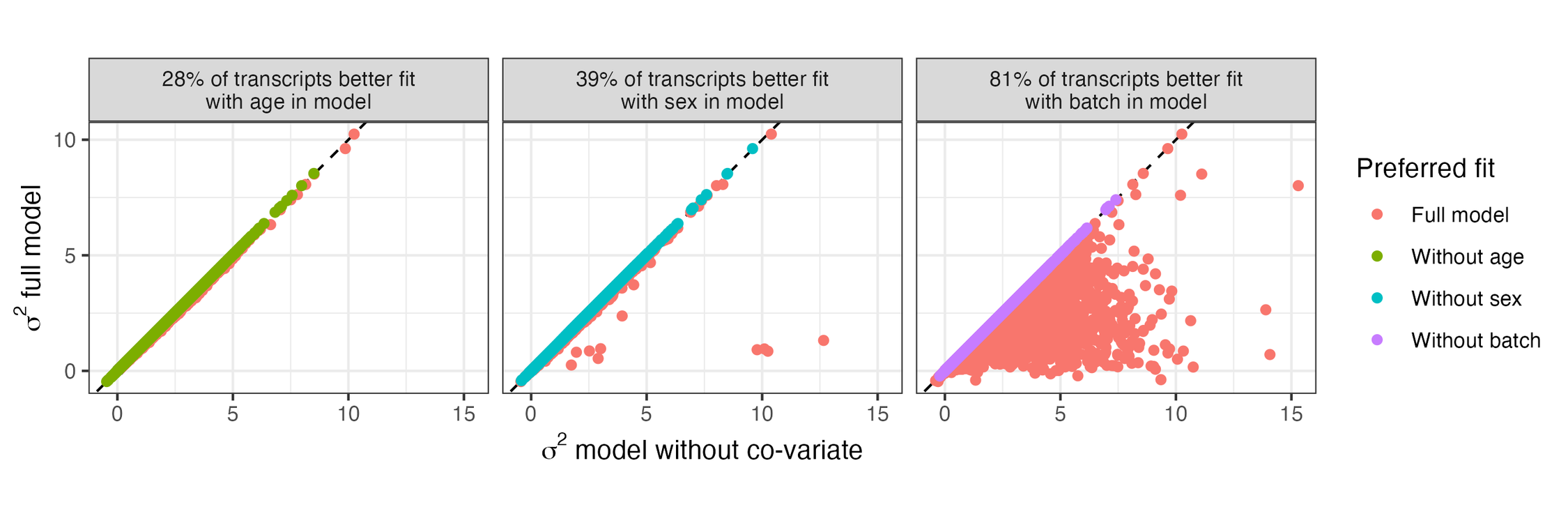

Supplement: S1 Fig — Sigma plots demonstrate improved fit for ~80% of transcripts when the model is adjusted for sequencing batch, whereas only 28% and 37% of transcripts have improved fit when the model is adjusted for age and sex, respectively. All three adjustments were applied to the final model. (TIF) [file pone.0284498.s001.tif]

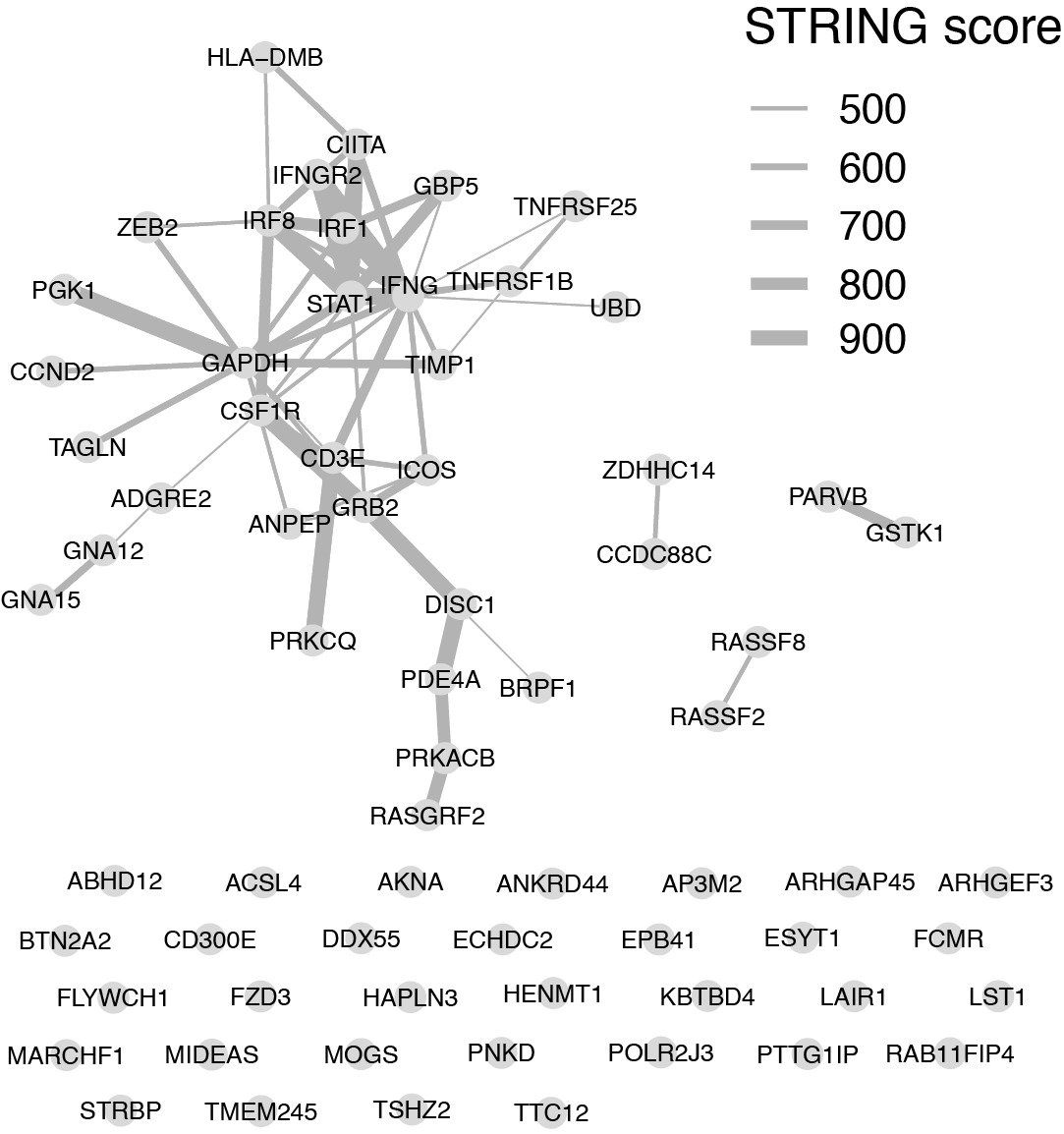

Supplement: S2 Fig — Genes (n = 67) with ≥1 differentially expressed transcript (DET, FDR <0.05) either from the media (n = 2 DETs) or Mtb-stimulated condition (n = 77 DETs) were analyzed by STRING (string-db.org) using known interactions (experimentally determined or derived from curated databases), co-expression and text mining. One gene (AC055839.2) did not map to STRING. Nodes represent genes. Edges are combined scores >400 (medium confidence) with thickness indicating score value. (TIF) [file pone.0284498.s002.tif]
